# Radiofrequency ablation versus resection for technically resectable colorectal liver metastasis: a propensity score analysis

**DOI:** 10.1186/s12957-018-1494-3

**Published:** 2018-10-15

**Authors:** Li-Jun Wang, Zhong-Yi Zhang, Xiao-Luan Yan, Wei Yang, Kun Yan, Bao-Cai Xing

**Affiliations:** 1 Key laboratory of Carcinogenesis and Translational Research (Ministry of Education/Beijing), Department of Hepatopancreatobiliary Surgery Unit I, Peking University Cancer Hospital and Institute, 52 Fucheng Road, Haidian District, Beijing, 100142 China; 2 Key laboratory of Carcinogenesis and Translational Research (Ministry of Education/Beijing), Department of Ultrasound, Peking University Cancer Hospital and Institute, 52 Fucheng Road, Haidian District, Beijing, 100142 China

**Keywords:** Radiofrequency ablation, Resection, Liver metastasis, Colorectal cancer, Survival

## Abstract

**Background:**

Liver resection is the first-line treatment for patients with resectable colorectal liver metastasis (CRLM), while radiofrequency ablation (RFA) can be used for small unresectable CRLM because of disease extent, poor anatomical location, or comorbidities. However, the long-term outcomes are unclear for RFA treatment in resectable CRLM. This study aimed to compare the recurrence rates and prognosis between resectable CRLM patients receiving either liver resection or RFA.

**Methods:**

Consecutive patients who underwent RFA or hepatic resection from November 2010 to December 2015 were assigned in this retrospective study. Propensity score analysis was used to eliminate baseline differences between groups. Survival and recurrence rates were compared between patients receiving liver resection and RFA.

**Results:**

With 1:2 ratio of propensity scoring, 46 patients in the RFA group and 92 in the resection group were successfully matched. Overall survival was similar between the two groups, but the resection group had a higher disease-free survival (median, 22 months vs. 14 months). Whereas among patients with a tumor size of ≤ 3 cm, disease-free survival was similar in the two groups (median, 24 months vs. 21 months). Compared to the resection group, the RFA group had a higher rate of intrahepatic recurrence (34.8% vs. 12.0%) and a shorter recurrence free period. The local and systemic recurrence rate and recurrence-free period for the same were insignificant in the two groups. Poor disease-free survival was associated with RFA, T4, tumor diameter > 3 cm, and lymph node positivity.

**Conclusion:**

Among patients with technically resectable CRLM, resection provided greater disease-free survival, although both treatment modalities provided similar overall survival.

## Background

Liver metastasis is the leading cause of cancer-related mortality in patients with colorectal cancer [1, 2]. Approximately 50% of patients with colorectal cancer develop liver metastases, with 15–25% have it at their diagnosis [3, 4], with 35% at stage IV disease at presentation, and 20 to 50% with stage II or III disease progress to stage IV [5]. Surgical resection remains the gold standard for treating colorectal liver metastases (CRLM) and can cure some patients or substantially prolong their survival. Recent 5-year survival rates are 30–50% as reported [6–9]. However, most patients are not initially candidates for resection because of disease extent, anatomical location, or comorbidities [10–13]. In addition, concerns regarding complications and mortality have limited the use of resection.

Radiofrequency ablation (RFA) is a widely used minimally invasive modality that provides acceptable local control for small tumors [14, 15] and may be an alternative for treating unresectable CRLM. The European Society for Medical Oncology guidelines for metastatic colorectal cancer recommends RFA with surgery to achieve R0 resection or as a liver-preserving alternative to resection in cases of poor anatomical localization [16]. An international panel of ablation experts has also reached a consensus regarding the use of thermal ablation for CRLM [17].

Previous research indicate RFA as inferior to resection in treating liver metastases > 3-cm tumor size [18, 19]. However, improvements in RFA have facilitated the ablation of a spherical zone with a diameter of > 5 cm [20, 21], which has enhanced its applicability. Nevertheless, it remains unclear whether the long-term outcomes of RFA are comparable to those of hepatic resection for resectable CRLM, and so far, no randomized controlled trial has been published. Furthermore, retrospective studies may be limited by patient selection bias and publication bias, although propensity score matching analysis has been successfully used to minimize bias in retrospective studies [22, 23]. Therefore, the present study compared the recurrence and survival rates for RFA and hepatic resection among patients with technically resectable CRLM using propensity score analysis.

## Methods

### Study design, selection of patients, and grouping

This retrospective study evaluated collected data from 428 consecutive patients who underwent RFA or resection for CRLM at the Peking University Cancer Hospital between November 2010 and December 2015. The study was approved by the Clinical Research Ethics Committee of the same hospital and was performed in compliance with the Helsinki Declaration. Written informed consent was obtained from all patients.

Inclusion criteria was patients with ≤ 3 tumors, well-located tumor size of ≤ 5 cm, and absence of uncontrolled extrahepatic disease. The exclusion criteria were patients with recurrent CRLM after previous resection or RFA, or who underwent both RFA and resection in one session, and those who received palliative treatment. The patients’ preoperative images were retrospectively viewed to confirm the technically resectable disease CRLM which was feasibility of complete macroscopic resection to maintain at least 30% future liver remnant [24]. Based on these criteria, we included 50 patients who received RFA and 160 patients who underwent resection with curative intent.

### Study outcomes

Baseline data included sex, age, timing of metastasis, location of primary cancer, T stage and N stage, number and diameter of hepatic metastases, carcinoembryonic antigen (CEA) level, and neoadjuvant chemotherapy in the two groups. Disease-free survival and overall survival was determined in both the groups. Variables between the two groups and those included in clinical risk score that could have impacted on survival were identified.

**Fig. 1:**
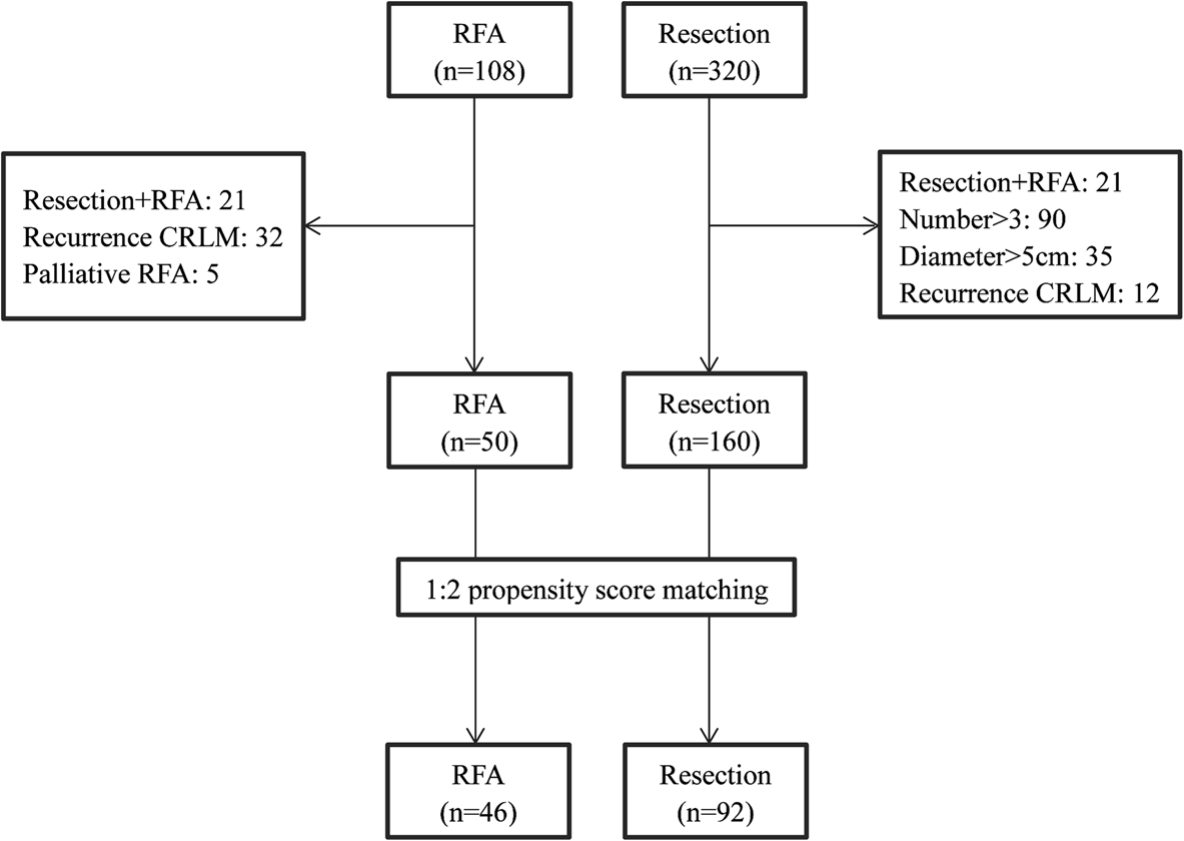
Flow chart of the study

The propensity scores were estimated using a logistic regression model that included the following five covariates primary lymph node status, synchronicity, number of metastases, size of the largest metastasis, and preoperative CEA levels. A 1:2 “nearest neighbor” match paradigm was used. Patients were matched using a caliper of 0.15 in each group (Fig. 1).

### Hepatic resection

The liver was examined, and intraoperative ultrasonography was performed to identify the number and locations of metastases. The extent of hepatic resection was determined by the number, diameter, and locations of the tumors, and lobectomy, segmentectomy, or limited resection was adopted. Parenchymal dissections were performed using the clamp method with Peng’s multifunctional operative dissector (Hangzhou Shuyou Medical Instrument Co., Ltd., PR China; FDA 510[K] number K040780). An intermittent Pringle’s maneuver with clamping of the hepatoduodenal ligament was occasionally performed during parenchymal transection for vascular occlusion. The preserved margin during parenchymal dissection was ≥ 5 mm.

### Radiofrequency ablation

The indications for RFA were complete necrosis achieved based on the tumor size and its position, patients’ comorbidities that precluded general anesthesia or surgery, and patient choice. RFA was more often used in deeply situated tumors that would have required excessive sacrifice of the normal parenchyma in resection. Ablation of tumors next to major bile ducts (common bile duct, common hepatic, right and left hepatic ducts) within 1 cm, in contact with larger blood vessels (portal vein and hepatic vein), or in close proximity to vulnerable structures (colon, gallbladder etc.) were relatively restricted. All RFA procedures were performed using the Celon system (Teltow, Germany) by radiologists with > 5 years of interventional experience. The bipolar electrode needles were 16G, and scanning/guidance ultrasonography was performed using the Aloka α-10 (Tokyo, Japan) and GE Logiq E9 (Connecticut, USA) devices. Electrodes were inserted into the tumor under ultrasonographic guidance, and overlapping ablations were used for > 3-cm tumors. The ablation end-point was determined based on the impendence and output power, as well as coverage of the safety margins. Track ablation was performed after the treatment. The ablative area appeared hyperechoic on ultrasound during RFA procedure, which should cover the tumor area. For cases of difficult to assess, contrast-enhanced ultrasound was performed immediately after RFA. If tumor residual occurred, additional RFA session was performed.

### Follow-up and definition of recurrence

Patients were evaluated by contrast-enhanced computed tomography (CECT) or magnetic resonance imaging (MRI) at 1 month after resection or RFA procedure. Then, CEA test, MRI of the abdomen, CT of the chest, and MRI or CT of the pelvis were repeated every 3 months for 2 years and every 6 months thereafter. Recurrences were typically identified radiologically.

Local recurrence was defined as tumor growth at the treatment site. Intrahepatic recurrence was defined as new liver lesions emerging at a non-treatment site. Systemic recurrence was defined as tumors at both hepatic and extrahepatic sites, including recurrence at the site of the primary tumor.

### Statistical analysis

Continuous variables were reported as median and interquartile range. Inter-group differences were analyzed using the chi-square test, Fisher’s exact test, or Student’s *t* test, as appropriate. Survival data were analyzed using the Kaplan-Meier method and the log-rank test. Variables with a univariate *p* value of < 0.1 were entered into the Cox regression model for multivariate analysis. A *p* value of < 0.05 was considered statistically significant.

## Results

### Clinicopathological characteristics

The resection group included 92 patients (58 males, 34 females) with a median age of 63 years (interquartile range 51.0–65.8), and the RFA group included 46 patients (29 males, 17 females) with a median age of 63 years (interquartile range 50.8–67.0). The patients’ clinicopathological characteristics are shown in Table 1. After matching according to the propensity score, there was no significant difference between the two groups although differences were originally observed for preoperative CEA levels and the number, size, and location of the liver metastases. The 46 patients in the RFA group underwent treatment for 55 lesions (1.2 ± 0.5 lesions/patient), and the 92 patients in the resection group underwent treatment for 114 lesions (1.2 ± 0.4 lesions/patient). The median diameter in the RFA group was 2.3 cm (range, 1.7–3.6 cm), compared to 3 cm (range, 1.9–3.6 cm) in the resection group. Thirty-four patients (37.0%) in resection group and 22 patients (47.8%) in the RFA group received neoadjuvant chemotherapy. Patients in the two groups received regular systemic chemotherapy regimens, such as FOLFOX, CAPEOX, or FOLFIRI, combining biologic-targeted agents (bevacizumab or cetuximab) which were selectively used in high risk of recurrence patients only. After treatment, 45 (48.9%) patients in the resection group and 16 (34.8%) patients in the RFA group received adjuvant chemotherapy according to preoperative chemotherapy response, Fong’s score, and postoperative recovery condition, and the difference was not statistically significant (*P* = 0.115).

**Table 1:** The patients’ demographic and clinical characteristics

Characteristics	Surgery (*n* = 92)	RFA (*n* = 46)	*P* value
Sex			1.000
Male/female	58/34	29/17	
Age (years)	58.0 (51.0–65.8)	58.5 (50.8–67.0)	0.492
Preoperative CEA (ng/mL)	6.7 (2.9–22.3)	5.4 (3.2–12.9)	0.731
Location of primary cancer			0.802
Colon/rectum	58/34	30/16	
Timing of metastasis			0.277
Synchronous/metachronous	70/22	31/15	
T stage			0.798
T4/T1–3	30/62	16/30	
N stage			0.899
N0/N+	31/61	16/30	
Median diameter (mm)	30.0 (18.5–35.8)	22.5 (16.8–36.3)	0.249
No. of tumors			0.878
1/2–3	75/17	37/9	
Location of liver metastasis			0.076
Unilobar/bilobar	73/19	42/4	
Neoadjuvant chemotherapy			0.220
Yes/no	34/58	22/24	
Extrahepatic disease			0.160
Yes/no	4/88	5/41	
Comorbidities			0.232
Hypertension	14	5	
Diabetes	8	1	
Cardiac	5	3	
Cerebrovascular	5	2	
Pulmonary or others	2	4	

*CEA* carcinoembryonic antigen, *RFA* radiofrequency ablation

### Survival analysis

**Fig. 2:**
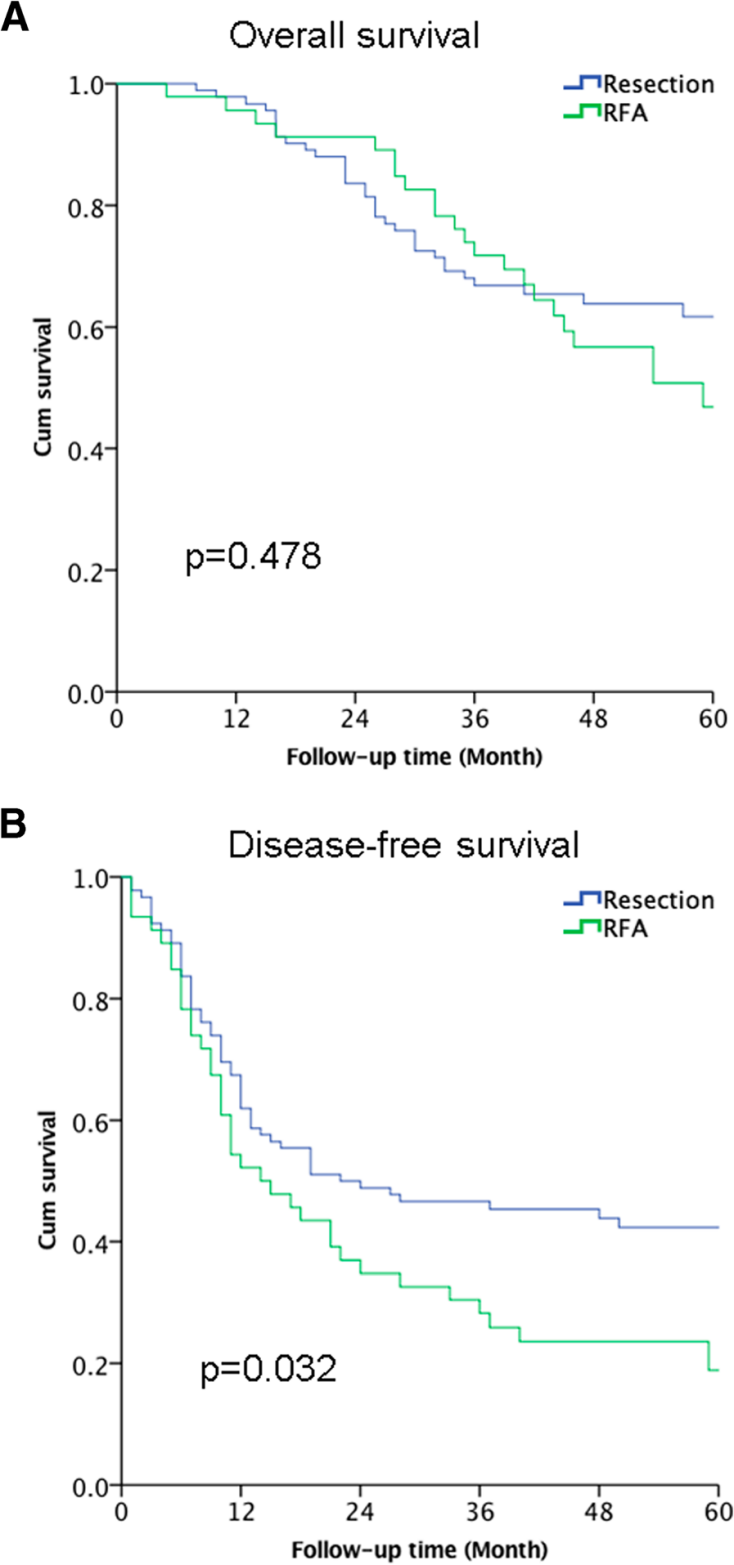
Overall survival (**a**) and disease-free survival (**b**) for patients who underwent radiofrequency ablation (RFA) or hepatic resection after matching

All follow-ups ended in July 2018, and the median follow-up was 44 months (range, 6–96 months). The overall survival (OS) rates were similar in the resection and RFA groups at 1 year (97.8% vs. 95.7%), 2 years (83.6% vs. 91.3%), and 3 years (66.8% vs. 71.6%). Based on the Kaplan-Meier analyses, the median OS was 74 months in the resection group and was 59 months in the RFA group (*P* = 0.484, Fig. 2a). The median disease-free survivals (DFS) were 22 months after resection and 14 months after RFA (*P* = 0.032, Fig. 2b). However, the DFS for resection and RFA were similar among patients with a tumor size of ≤ 3 cm (24 months vs. 21 months, *P* = 0.41).

### Recurrence and treatment

The first sites of disease progression after treatment are shown in Table 2. Intrahepatic recurrence was significantly common (36.9% vs. 11.9%, *P* = 0.001), and local recurrence was more common in the RFA group (15.2% vs. 6.5%, *P* = 0.099) (Table 2). The systemic recurrence rates were similar in both groups (26.1% vs. 39.1%, *P* = 0.129). Hepatic recurrence was more common after RFA compared to resection (69.6% vs. 32.6%, *P* < 0.001) (Table 2).

**Table 2:** Recurrence after treatment using RFA or surgery and the subsequent treatment

Recurrence	Surgery (*n* = 92)	RFA (*n* = 46)	*P* value
First recurrence pattern	53	36	
Local recurrence	6	7	0.099
Intrahepatic recurrence (de novo)	11	17	< 0.001
Systemic recurrence	36	12	0.129
Hepatic recurrence			0.001
Yes	30	32	
No	62	14	
Treatment for first recurrence			0.089
Curative treatment	17	18	
Resection		5	
RFA	2	11	
Resection + RFA	1	1	
Radiotherapy	3	1	
Resection + radiotherapy	2	0	
Palliative treatment	36	18	
Chemotherapy	27	15	
Best supportive care	9	3	

*RFA* radiofrequency ablation

**Fig. 3:**
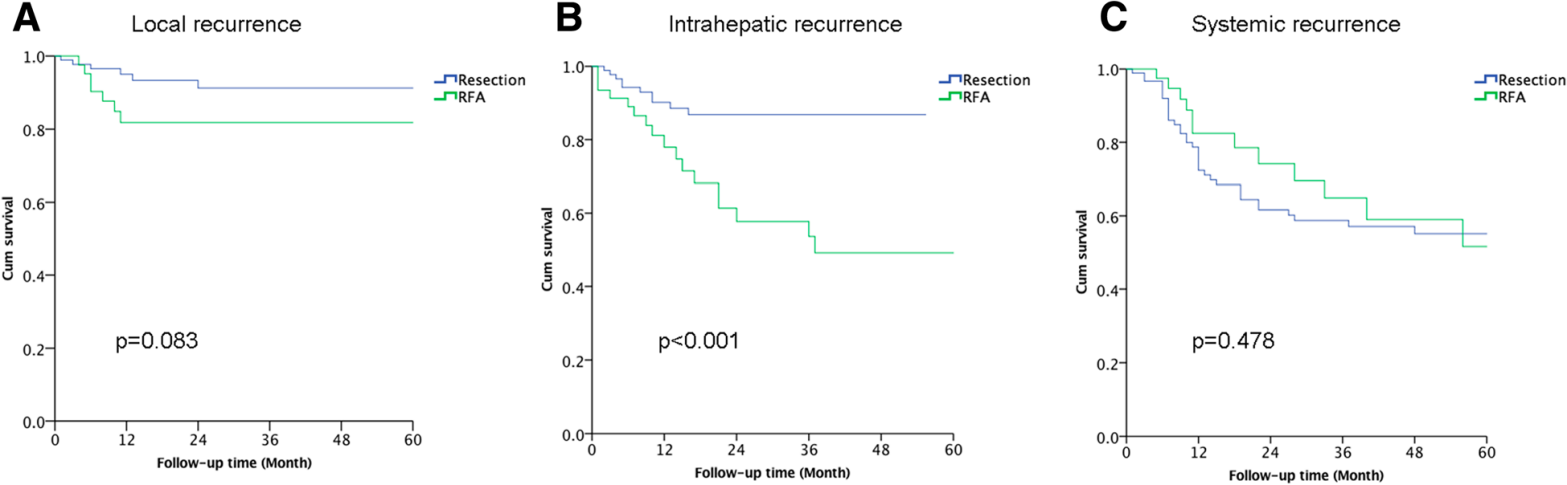
Times to local recurrence (**a**), intrahepatic recurrence (**b**), and systemic recurrence (**c**) among patients who underwent radiofrequency ablation (RFA) or hepatic resection for colorectal liver metastases after matching

The time to local, intrahepatic, and systemic recurrences are shown in Fig. 3. The RFA group had a significantly shorter time to intrahepatic recurrence, compared to the resection group (*P* < 0.001). No significant differences were observed between the two groups for the times to local recurrence (*P* = 0.083) or systemic recurrence (*P* = 0.478). Additional treatments with curative intent (resection, RFA, radiotherapy, or combination therapy) were performed after recurrence for 18 patients (50.0%) in the RFA group and 17 patients (37.0%) in the resection group (*P* = 0.089).

### Multivariate analyses of DFS and OS

Cox multivariate analyses were used to evaluate DFS, and the results revealed that poorer DFS was independently associated with RFA, T4 status, lymph node positivity, and tumor diameter > 3 cm (Table 3). OS was independently associated with tumor diameter > 3 cm and T4 stage, but was not significantly associated with RFA or resection as first-line treatment.

**Table 3:** Multivariable analyses of disease-free survival and overall survival

Characteristics	Number	Risk ratio	95% CI	*P* value
Disease-free survival				
Sex (male/female)	87/51	1.338	0.859–2.085	0.197
RFA/resection	46/92	1.661	1.085–2.543	0.020
T stage (T4/T1–3)	46/92	1.652	1.059–2.579	0.027
N stage (N+/N0)	91/47	1.872	1.163–3.014	0.010
Diameter (> 3 cm/≤ 3 cm)	52/86	2.315	1.504–3.564	< 0.001
Overall survival				
RFA/resection	46/92	1.198	0.453–1.778	0.494
T stage (T4/T1–3)	46/92	2.152	1.293–3.583	0.003
Diameter (> 3 cm/≤ 3 cm)	52/86	1.925	1.156–3.206	0.012
Adjuvant chemotherapy (no/yes)	77/61	1.460	0.523–1.460	0.608

*RFA* radiofrequency ablation, *CI* confidence interval

## Discussion

Hepatic resection is the first-line treatment for patients with resectable disease and may provide a cure or survival benefit [6, 10]. However, RFA has emerged as a less invasive alternative that has a lower complication rate and shorter hospital stays [25, 26]. RFA is effective for unresectable CRLM among patients with comorbidities and recurrent liver disease and may be added to surgery to increase the chance of curative resection and improve survival rates [14, 16, 27].Nevertheless, design challenges have prevented researchers from performing randomized controlled trials to compare RFA and resection among patients with resectable CRLM. Furthermore, the retrospective studies of RFA versus resection for resectable CRLM have been limited by imbalances in the lesion and patient characteristics [28, 29], although propensity score analysis can be used to address these issues in retrospective studies. Previous studies have suggested that tumor diameter and number are the most important factors that influence the effect of RFA, although primary lymph node status, timing of metastasis, and CEA levels can also influence patient survival and recurrence [30–33]. To prevent selection bias towards RFA, we analyzed multiple clinicopathological characteristics to identify inter-group differences and were able to create propensity score-matched groups of patients who underwent RFA or resection for CRLM.

The Kaplan-Meier analysis revealed that the RFA group had shorter DFS and more patients who experienced hepatic recurrence, compared to the resection group. Thus, it is important to understand if patients were harmed by including them in the RFA treatment protocol. DFS outcomes were similar in both the groups for tumor diameter of ≤ 3 cm, demonstrating that the best indication for RFA were patients with resectable CRLM having ≤ 3-cm tumor diameter.

Recent studies have reported local disease progression rates of 9–48% for percutaneous RFA, compared to 2–9% for resection [34–36]. Evaluation of local recurrence patterns and time to recurrence demonstrated the treatment efficacy of resection over RFA. This relatively high local failure rate in the RFA group could be related to incomplete ablation of larger lesions, the heat sink effect, and/or treatment modality-specific limitations. Interestingly, the de novo intrahepatic recurrence was significantly shorter for the RFA group. This finding may have several explanations. Firstly, previous studies have demonstrated that additional unidentified liver metastases may be revealed during surgical exploration, which would not be treated using percutaneous RFA [37–39]. In the present study, 5.4% of patients had initially undetected liver metastases that were identified during the surgery. Secondly, the RFA group had a relative lower proportion of patients who received adjuvant chemotherapy, compared to the resection group, which was related to their comorbidities, unwillingness to receive adjuvant chemotherapy, and other reasons. Thirdly, RFA may contribute to the dissemination of tumor cells and may induce immunological processes that favor tumor growth [34, 40], although we cannot exclude the possibility of resection accelerating the growth of new lesions [29, 41]. Similar to the findings of previous studies, we observed that both groups had similar rates of systemic metastases.

The prolonged survival that we observed in the present study may be related to treatment selectivity, as approximately 25% of patients experience locoregional recurrence after RFA or resection for CRLM [41]. In addition, repeated hepatic resection and RFA are associated with long-term survival and possible cure [21, 42], although resection should be performed if the extrahepatic metastases can be completely removed [43]. In the present study, patients with recurrence underwent a comprehensive assessment and then received curative or palliative chemotherapy according to the recurrence pattern. Survival analysis revealed that repeated curative treatment increases the likelihood of long-term survival among patients with recurrent colorectal metastases.

Several studies have reported conflicting results regarding whether RFA is inferior or equivalent to resection among patients with resectable colorectal disease [34, 44, 45]. However, these studies were limited by selection bias, as the groups were not equivalent. Previous studies have also reported varying 3-year survival rates in both treatment groups. For example, Oshowo et al. [25] reported 3-year OS rates of 55.4% for hepatic resection and 52.6% for RFA, while Otto et al. [46] reported 3-year OS rates of 67% for hepatic resection and 60% for RFA. The 3-year survival rates in the present study were similar for both treatment groups (66.8% for resection vs. 71.7% for RFA). OS rates in this study are higher than the rates from previous studies, which may be related to our patient selection criteria based on the European Society for Medical Oncology consensus (oligometastatic disease with relatively less invasive behavior). Although RFA provided inferior DFS in the present study, the multivariate analysis did not reveal any significant difference in OS. This finding is partially related to the frequency of curative therapy after recurrence in the RFA group (50% vs. 37%). Another reason is that the follow-up period is short (median 44 months) and it is likely that OS superiority is not reached in the resection group. Thus, larger studies are needed to provide more reliable evidence regarding this association.

The present study has several limitations. First, we used a retrospective design and the patients could not be randomized, as the two groups had different burdens of disease and oncological statuses, although a propensity score-based analysis cannot account for the effects of variables that were not analyzed. Second, the sample size was relatively small because we only considered patients with resectable disease. Third, the RFA group had a smaller proportion of patients who received perioperative chemotherapy, which is likely related to the RFA group including patients with more severe comorbidities, patients who were unwilling to receive chemotherapy, and/or patients with treatment selection bias. Thus, the association of perioperative chemotherapy with poorer DFS in the RFA group should not be ignored.

The strength of the study lies in the propensity score-based analysis used to overcome the effects of potential confounders, the Cox multivariable analysis for DFS, and OS; all these performed for the small sample size to arrive at a conclusion for RFA and resection as treatment options for colorectal metastases.

## Conclusions

In conclusion, hepatic resection provided superior DFS, compared to RFA, among patients with technically resectable CRLM. However, multivariate analysis did not reveal any significant treatment-related differences in OS between the RFA and resection groups.

## Abbreviations

CEA: Carcinoembryonic antigen
CI: Confidence interval
CRLM: Colorectal liver metastasis
DFS: Disease-free survivals
OS: Overall survival
RFA: Radiofrequency ablation
